# Dehydration kinetics of nanoconfined water in beryl probed by high temperature single crystal synchrotron X-ray diffraction

**DOI:** 10.1038/s41598-024-53654-4

**Published:** 2024-03-13

**Authors:** Phuong Q. H. Nguyen, Dongzhou Zhang, Jingui Xu, Robert T. Downs, Przemyslaw K. Dera

**Affiliations:** 1 Hawaii Institute of Geophysics and Planetology, University of Hawaii at Manoa, Honolulu, HI 96822 USA; 2 GeoSoilEnviroCARS, University of Chicago, Argonne, IL 60439 USA; 3 Key Laboratory for High-Temperature and High-Pressure for the Earth’s Interior, Institute of Geochemistry, Chinese Academy of Sciences, Guiyang, China; 4 Department of Geosciences, The University of Arizona, Tucson, AZ 85721-0077 USA

**Keywords:** Chemical bonding, Geophysics, X-ray diffraction, Structural properties

## Abstract

Understanding changes in material properties through external stimuli plays a key role in validating the expected performance of materials and engineering material properties in a controlled manner. Here, we introduce a fundamental protocol to deduce dehydration reactions kinetics of water confined in nanopore channels, with the cyclosilicate beryl as the scaffold of interest, using time-resolved synchrotron X-ray diffraction (SXRD), in the temperature interval of 298–1038 K. The temperature-dependent intensity $(I)$ of the strongest reflection (112) was used as the crystallite variable. An estimation of an isobaric thermal crystallite coefficient, $k$, analogous with the isobaric thermal expansion coefficient, established the rate of relative crystallization as a function of temperature, $\frac{\partial I}{\partial T}$. A plot of $lnk$ and $\frac{1}{T}$ gives rise to two kinetic steps, indicating a slow dehydration stage up to ~ 700 K and a fast dehydration stage up to the investigated temperature 1038 K. The crystal structure of beryl determined up to 1038 K, in temperature increment as small as 10 K, indicates the presence of channel ions Na and Fe and a gradual decrease of water upon heating.

## Introduction

The nature of fluid–solid interactions is a subject that evolved considerably in its scope in both geology and material science. A crucial subset of the subject is the analysis of dynamics and structure of water, or other fluids, in confined systems. Characterizing the behavior of water confined in such materials is crucial to advance the understanding of macroscopic phenomena, i.e. ion exchange/mobility, ultrafiltration, adsorption, and in turn serves as a guideline for specifically molecular engineered purposes^1–5^. It is well-accepted that the properties of nanoconfined water is substantially different from what is commonly observed in bulk water^6^.

Within the geological context, the majority of chemical reactions near the Earth’s surface involve fluid, e.g. aqueous phases and take place either at fluid–solid interfaces or in confined spaces of mineral interlayers and nanopores^7^. Cyclosilicates have attracted considerable interest due to their zeolite-like channels that contain a variety of large volatiles as molecular species (H_2_O and CO_2_, etc.), as well as ions.

Beryl, with ideal chemical composition Be_3_Al_2_Si_6_O_18_, is a common silicate mineral found in igneous and metamorphic rocks and has served as a minor ore of beryllium. Its differently colored varieties, including green emerald (due to Cr^3+^ substituting for Al), green beryl (V^3+^), blue aquamarine (Fe^2+^), yellow heliodor (Fe^3+^), pink morganite (Mn^2+^), and red bixbite (Mn^3+^) are among the most popular gemstones^8^. Beryl occurs mainly in granite pegmatites^9^ and sometimes in rhyolite, metamorphic rocks, or in the veins and cavities of limestones and marbles^10^. There are currently ten minerals with the beryl structure, including the geologically and industrially important mineral cordierite, (Mg,Fe)_2_Al_3_(AlSi_5_O_18_) that are characterized by hexagonally-packed open honeycomb rings of corner-sharing tetrahedra cross-linked by tetrahedral and octahedral groups. The tetrahedral rings of hexagonal beryl consist of six equivalent SiO_4_ tetrahedra, whereas those in orthorhombic cordierite have four SiO_4_ and two AlO_4_ tetrahedra. According to the Al-avoidance rule^11^, the two Al^3+^ ions occupy opposite sites in the ring in ordered cordierite, lowering the symmetry to orthorhombic. The rings are stacked along the [001] direction forming channels that pinch to bottlenecks ~ 2.8 Å or swell to large cages ~ 5.1 Å in diameter^12–15^. Consequently, there are two positions along the beryl channel axis where ions or absorbed species typically reside, corresponding to the swells at fractional atomic coordinates (0 0 0.25) and the pinches at (0 0 0), designated as Wyckoff positions 2a (C1) and 2b (C2), respectively.

Ideal beryl and cordierite are nominally anhydrous, but natural samples usually contain water and carbon dioxide in the channels, which is related to the fugacity of the crystallization environment^16^. Water within the structure of hydrous beryl has been classified as two different types, depending on the orientation of the C2 symmetry axis of the water molecule relative to the c axis of beryl, perpendicular as type I, common for unsubstituted or weakly substituted cationic beryl, and parallel as type II, dominant in rich cationic substituted beryl.

Many ring silicates exhibit unusual thermal expansion behavior. Cordierite, when heated to 800 °C expands along the a-axis, but contracts along the c-axis^17^. Beryl, when heated below ~ 300 °C, contracts along the c-axis, but expands at higher temperatures^18–20^. Cordierite has widespread industrial uses as a ceramic material, for instance as the substrate in catalytic converters, driving interest in its high temperature transformation. The industrial applications of beryl have been more limited, due to the toxicity of beryllium, but it is still a model system exhibiting a similar anomalous thermal response as cordierite. Beryl is also used as a diffraction calibrant for CheMin, the XRD instrument deployed on-board the NASA Curiosity Rover on the surface of Mars^21^.

One of the best sources of detailed information about the structural mechanisms of thermal expansion are in situ X-ray diffraction experiments at high temperature. Experimental capabilities for this kind of measurements are fairly standard on modern commercial diffractometers, but limited to about 500 K. On the one hand, custom setups that reach about 1000 K have been built over the last few decades, but measurements exceeding 500 K still remain state-of the-art, requiring significant data collection time (several hours) at each temperature and involve meticulous sample realignments at each temperature change, making it time consuming to cover a large temperature range in fine increments. On the other hand, discontinuous high temperature phenomena such as subtle phase changes, dehydration, or cation diffusion, often require fine T-steps to capture accurately. As part of the Partnership for eXtreme Xtallograpy project located at the GSECARS facility at Advanced Photon Source, Argonne National Laboratory, in experimental station 13BM-C, we developed a setup and measurement methodology for rapid synchrotron high temperature experiments. A complete high-quality single-crystal data collection on our instrument can be completed in under 1 min, and with the use of single crystal sapphire capillaries as sample mount, temperatures as high as 1400 K can be reached.

We elected beryl as the material of choice as it is a common Be-bearing mineral, in which low Z element such as Be continues to push the detection limit of conventional X-ray diffraction. Furthermore, the gem quality of beryl (especially its green variation emerald) as well as its well-defined channels with confined light elements, e.g. H_2_O, CO_2_, in its structure, provides a controlled nanoporous model, allowing us to monitor subtle structural changes such as atomic mobility in response to external stimuli. Spectroscopic methods, e.g. infrared and Raman, introduce strong thermal radiation backgrounds at high temperatures, and is therefore not suitable to quantify the water content in-situ at elevated temperatures. On the other hand, X-ray diffraction is unaffected by the thermal radiation, and is therefore more suitable for in-situ high temperature measurements. Within the scope of this study, we aim to iterate the response of the beryl structure to increasing temperature, deduce dehydration reaction kinetics of the water, and introduce a fundamental crystallographic approach in assessing water behavior confined within the nanopore channels, using time-resolved synchrotron X-ray diffraction.

## Experimental

### Sample preparation

The beryl sample was obtained with permission from the University of Arizona RRUFF collection (RRUFF id # R040002, University of Arizona Mineral Museum id # 15681) and is from Usakos in Erongo Mountains, in Namibia, Africa. The analysis of the elemental composition of the sample was conducted by Electron Probe Micro Analysis (EPMA) using a Camera SX50 operated at 15 kV and 20 nA with a 10 µm spot size at the University of Arizona and indicated a chemical formula (Be_0.97_Li_0.03_)_3_(Al_0.97_Fe^3+^_0.03_)_2_Si_6_O_18_·Na_0.01_ (Table S1). The large specimen was homogeneous, free from chemical zonation and twinning. Structural water content was not independently determined. For the high temperature experiments, a single crystal of the colorless natural beryl, ~ 100 × 50 × 50 µm^3^ in volume, was inserted into a single crystal quartz capillary with 200 µm inner diameter (ID) using a thin needle. A ~ 15 × 15 × 1 µm^3^ flake of gold was placed on top of the beryl single crystal and used as a temperature calibrant.

### Zoning potential in beryl

The starting beryl material comes from a large piece of crystal. We screened multiple smaller crystals and XRD patterns show similar results. If there were potential zoning, single crystal XRD from different fragments would not be consistent. Moreover, the XRD results show similar peak intensity, indicating no significant change in structure factor. Back-scattered electrons (BSE) image of a beryl sample grain used for EPMA measurements is provided in Figure S10. The sample appearance is uniform, with no visible gradients. There are some small-localized micro-inclusions visible, but they should not affect the type of measurements that is reported in our paper.

### Estimation of weighted water content in beryl channel

The beryl group of minerals shows quite significant chemical variability. The Mindat.com database defines a long list of possible impurities that can be integrated into the crystal, indicating Fe, Mn, Mg, Ca, Cr, Na, Li, Cs, O, H, OH, H_2_O, K, Rb. The specimens of beryl examined in our study come from a well-known locality in Usakos, Namibia, and represent one of the major/ most common types of beryl occurring at that locality, namely pale blue aquamarine. The chemical analysis results for the investigated beryl are consistent with the general chemical composition trends for aquamarines from Namibia. The work of Lum et al., which systematically examined water content in Namibian aquamarine beryls reported water concentrations, is consistent with the results of our crystallographic refinement, detailed in Section "Refinement of single crystal X-ray diffraction of beryl collected at ambient conditions" below^22^. Multiple models addressing the relationship between Na_2_O wt% and H_2_O wt% in emerald have been reported in the literature^23–26^. Here, we employed the model described by Henry et al., [0.6097 × Na_2_O wt%] + 1.6290 = H_2_O wt%, in which a relationship between Na_2_O wt%, obtained from EMPA data, and H_2_O in emeralds was suggested. The estimated water content in our beryl sample using this equation is 1.67 wt%.

### Refinement of single crystal X-ray diffraction of beryl collected at ambient conditions

The investigated beryl structure was solved with space group symmetry *P6/mcc*. Refined values of the unit cell parameters at 294 K were a = 9.2118(3) Å, c = 9.1955(3) Å and V = 675.76(6) Å^3^, which is in good agreement with the values a = 9.2122(2) Å, c = 9.1893(5) Å and 675.37(4) Å^3^, determined at the University of Arizona using high-resolution powder diffraction with a Cu source on a Bruker D8. Based on the c/a ratio and corresponding dominant type of cation substitution, beryl can be divided into tetrahedral, octahedral, and normal^27^. For our sample c/a = 0.9982, which identifies the cation substitution as “normal”. According to the elemental analysis, the extent of both tetrahedral and octahedral substitutions is minimal (below 3 at%). The only ions detected that depart from ideal end-member beryl formula were Li, Fe and Na, which are typical for natural beryl.

Most crystallographic studies agree that the silicon site in beryl is usually not affected by cation substitution. Attempts to refine the site occupancy of the silicon site freely resulted in a site occupancy factor 0.971, which was close enough to unity to assume ideal filling of this site with only silicon, and in the following refinements, we assumed Si occupancy factor is equal to 1.0 to maintain the charge balance.

EPMA suggests 0.06 apfu Fe is present in the beryl structure. Because of its ionic radius, iron is usually assumed to substitute in beryl in the octahedral aluminum site. Some earlier studies considered presence of iron in the channel sites. For example, Brown and Mills located the iron cations in hydrous alkali-rich beryl to be present at the 2b channel site, with the octahedral site also completely filled with Al^14^. Goldman et al.^9^ and Wang et al.^28^ identified spectroscopic features in the electronic absorption spectra that are uniquely caused by Fe^2+^ present in the beryl channels^29^. In refinements that excluded any extra-framework species, two strong Q peaks were discovered in the difference Fourier maps, located at the 2a and 2b channel positions. An attempt at filling the 2b site exclusively with sodium cations resulted in site occupancy factor 0.09, far exceeding the sodium content determined by EPMA, and suggesting the presence of species with higher electron number. We first set out to model Fe in our sample being present exclusively in the 2b channel site. The refinement with Fe2b unconstrained and no Na present resulted in site occupancy factor 0.038, below the EPMA result of 0.06. This suggested either (1) the presence of an element with lower electron density than Fe or (2) Fe ions are present in more than just the 2b channel position. With occupancy factor Na2b set to the EPMA value, we probed a stable model of Fe ions that were being distributed in the octahedral site of Al and 2b channel site. EPMA suggests 0.09 apfu Li present, a stable crystallographic model was reached upon incorporating Li^+^ in the beryllium tetrahedral site.

The nature of structural water present in the channels of the beryl structure has been a topic of intensive investigation using spectroscopic, computational and crystallographic methods. Determination of hydrogen positions using X-ray diffraction methods is challenging due to the low atomic scattering factor, particularly when disorder and fractional occupations are involved. To date only 4 papers reported hydrogen atom locations in beryl^30–33^, with the most accurate data determined from the neutron diffraction experiments^30,31^. Artioli et al. determined the fractional coordinates of hydrogen atoms of 2a water molecule in morganite beryl to be (0.072 0.090 0.318), which corresponds to hydrogen disorder between 12 symmetry-equivalent positions^31^. A similar model was used in the crystalographic studies of alkali-poor beryl^32^ and Fe-rich dark-blue aquamarine^33^. In the same neutron diffraction study by Artioli et al., an alternative hydrogen atom location for 2a water molecule in aquamarine beryl was determined to be (0 0 0.165) corresponding to hydrogen disorder between 2 symmetry-equivalent positions^31^.

In our refinements, water was placed on the 2a channel site and was the only species occupying that position (Na and Fe ionic radii are not compatible with the 2a site). Final refinement resulted in O2a site occupancy factor 0.730. We also observed a maximum in the difference electron density map approximately 1 Å from the water oxygen site, displaced along the [001] direction, consistent with the earlier neutron diffraction study^31^. Fourier map indicating distribution of difference electron density (F_obs_-F_calc_) around the oxygen atom of the water molecule in the channel at T = 294 K is provided in Figure S12. In this refinement, Na, located at (0 0 0) had a partial occupancy of 7.9% and O, located at (0 0 1/4) from the water molecule inside the channel, had a partial occupancy of 68%. Hydrogen atoms of the water molecule in the channel were not included in the model. The highest difference Fourier peak (0.56e) was located along the sixfold axis, at position (0 0 0.1592), at a distance of 0.84 Å from the oxygen. These results are very similar to the model reported by Artioli et al. for their sample 2. The map was generated using WinGX ver. 2023.1 using 0.05 grid spacing resolution, and the figure was prepared using VESTA program^30,34,35^. The 3-dimensional isosurface maps were drawn with a cutoff of 0.04e. The small electron density peaks around the oxygen atom in the plane normal to the sixfold axis are below 0.05e.

### In situ synchrotron X-ray diffraction at high temperature

Figure S5 outlines the experimental setup, located at 13BM-C, under Partnership for eXtreme Xtallography program (PX2), hosted by GeoSoilEnviro Center for Advanced Radiation Source (GSECARS), Advanced Photon Source at Argonne National Laboratory. The capillary was mounted on a XYZ goniometer head, fixed on the φ rotational axis. The capillary was rotated from 0° to 340° at a rate of 0.5°/s for data collection. Each diffraction image covers a φ angle range of 0.5°, resulting in a total of 680 collected diffraction images for post analysis^36^. High purity nitrogen gas was introduced from the bottom of a double-walled alumina tube heater, passed over the tungsten coiled wire heating element, and covered the capillary in hot gas^37^. Resistive heating was achieved upon power supply up to ~ 275 W. A type-K thermocouple at the tube exit, positioned a few millimeters from the sample was used to measure the gas temperature. The power supply was remotely controlled via a feedback loop to achieve the desired temperature. The temperature measured by the thermocouple, based on feedback reading, was stable to within ± 1.5 K. Temperature increment was set to 10 K with the desired set temperature stabilized in < 5 s. At each set temperature, the gas flow heater was held for 1 min for the single crystal temperature to reach equilibrium. The precise temperature that the sample experienced during heating was determined using the thermal expansion of a gold flake placed on top of the sample^38^. A graph of temperature of hot gas recorded by the thermocouple placed at the tube exit against the temperature determined from the thermal expansion of gold flake is provided in Figure S11.

The X-ray spot size is 12 µm (H) × 18 µm (V) measured at full-width-at-half-maximum at a constant energy of 28.6 keV (0.434 Å). The experimental station consists of a 6-circle heavy diffractometer and Pilatus3 1 M photon-counting detector (Dectris). Auxiliary equipment includes a compact multipurpose optical table for accurate sample alignment.

The single-crystal diffraction data collection was controlled by the SPEC program (Certified Scientific Software) and has a Python-based user-interface. The UI provided calibration information for the Bruker APEX3 single-crystal diffraction data reduction software. The quality of data produced from the experiments were evaluated by the figures of merit of the resulting structural refinements, e.g. *R*_*1*_, *wR*_*2*_ and goodness of fit (GooF)^39^.

### Raman spectroscopy

Oriented Raman spectra were collected on a polished crystal surface using a custom-built Iris spectrometer system with a polarized 514 nm laser at a power of 100 mW for 80 s, and a SPEX HR460 nitrogen cooled detector. Raman spectra were collected on a randomly oriented prism-face of the crystal with a Thermo Almega microRaman system using a solid state 785 nm laser at a power of 500 mW and a thermoelectric cooled CCD detector. The laser is partially polarized with 4 cm^−1^ resolution and a spot size of 1 µm, collected 25 times for 10 s each. Additional data are available at https://rruff.info/R040002.

## Results and discussion

### Raman spectroscopy

**Figure 1:**
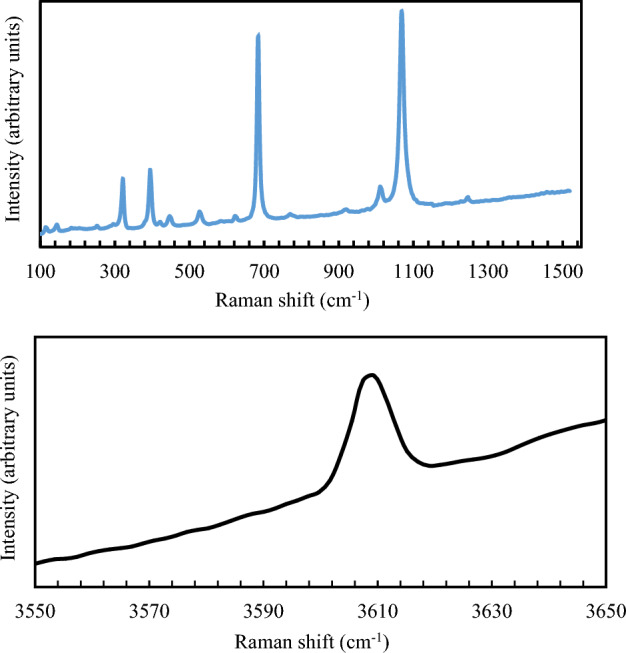
Raman spectra of investigated beryl. Laser is parallel to a* (100). Fiducial mark perpendicular to laser is parallel to c (001). Direction of polarization of laser is 45° counterclockwise relative to fiducial mark (top spectrum). Un-oriented sample in the bottom spectrum.

The arrangement of alkali and water molecules has been the subject of many spectroscopic studies^9,15,40–44^. These studies recognize three channel types related to different positions of alkali and water molecules in the channels, depending on the chemical composition of beryl: (1) alkali free; (2) Na-bearing (3) Li- and Cs-bearing. Two types of water molecules are connected with the presence/ absence of alkali ions: type I H_2_O, where the molecule symmetry axis is perpendicular to the L6 six-fold c axis of beryl, exists in alkali-free beryl; type II H_2_O, where the molecule symmetry axis is parallel to L6 and connects to the channel-bearing alkali ions. Both types of water exhibit characteristic vibrational modes—ν_1_ symmetric stretching, ν_2_ bending, and ν_3_ asymmetric stretching, which are all infrared and Raman active^45^. In this study, Raman spectra were collected and analyzed (Figs. 1, S8, S9). A summary of the assigned absorption bands, based on reported literature values, are provided in Table S7^9,40,46–57^. Besides the characteristic Raman active bands of the beryl framework in the region 200–1400 cm^−1^, we observed a single band at 3609 cm^−1^, assigned to the ν_1_ stretching vibrations H–O–H type-I of the water molecules in the channel. This observation is comparable with the low alkali content (< 0.08 wt%) beryl classification suggested by Lodzinski et al., where only a single Raman active band occurs at ca. 3609 cm^−1^ ^52^.

### In situ synchrotron XRD

**Figure 2:**
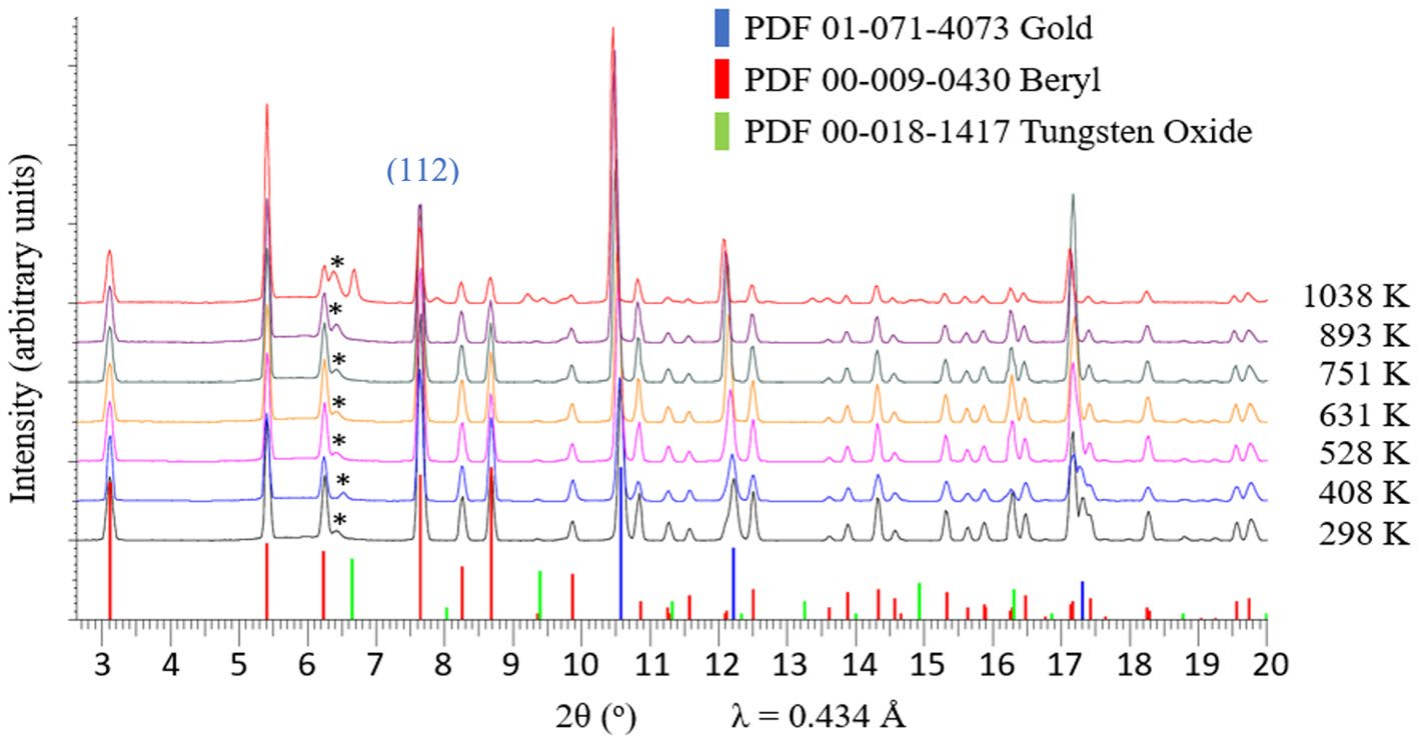
Selective SXRD spectra of beryl crystal at different temperatures. *X-Ray alignment pinhole sleeve from experimental setup.

A stack of selective XRD-patterns given in Fig. 2 presents the evolution of beryl as a function of temperature. Phase identification of experimental diffracted pattern of beryl matches well with reference card PDF 00-009-0430 together with the internal temperature calibrant gold, PDF 01-071-4073^58^. A dehydrated product was observed at > 893 K with a new set of prominent diffraction peaks showing up at 2θ of 9.21° and 9.82°. Another set of diffraction peaks, identified as WO_3_, PDF 00-018-1417, appeared at 1038 K, due to the oxidation of the coiled tungsten wire from the gas flow heater, coated on the outer wall of the quartz capillary. Thermal lattice expansion is expected as diffracted patterns shift toward lower 2θ angle with increasing temperature.

The lattices parameters determined on heating are fit with three different models for the temperature dependence of thermal expansion coefficients. To the first approximation, correlation of unit-cell parameters of beryl as a function of temperature can be fit by a least-squares method to:

1 $$V(T)={V}_{0}{e}^{\alpha (T-{T}_{0})}$$

with derived volume and axial thermal expansion coefficients as ${\alpha }_{V}=6.632\left(1\right)\times {10}^{-6} {K}^{-1}$, ${\alpha }_{a}=2.724\left(1\right)\times {10}^{-6} {K}^{-1}$, ${\alpha }_{c}=1.168\left(1\right)\times {10}^{-6} {K}^{-1}$, indicates that the thermal expansion is anisotropic (Fig. S6). We further employ the Berman et al. model (Eq. 2), taken into account that thermal expansion varies with T in a non-linear way, attributed to changes in the population and amplitude of vibrational modes within crystal^59,60^,

2 $$V\left(T\right)={V}_{0}(1+{a}_{1}\left(T-{T}_{0}\right)+{a}_{2}{\left(T-{T}_{0}\right)}^{2})$$

with first derivative giving rise to ${\alpha }_{V,T}={a}_{1}+2{a}_{2}(T-{T}_{0})$. Applying the experimental beryl volume data gives ${\alpha }_{V,298K}={a}_{1}=4.313\left(1\right)\times {10}^{-6} {K}^{-1}, {a}_{2}=4.748\left(5\right)\times {10}^{-9} {K}^{-2}$ and ${\alpha }_{V,1038K}=1.133\left(1\right)\times {10}^{-5} {K}^{-1}$. Corresponding axial thermal expansion coefficients for this model are ${\alpha }_{a,298K}= 1.848\left(5\right)\times {10}^{-6}, {a}_{2}=1.788\left(1\right)\times {10}^{-9}$ and ${\alpha }_{c,298K}= 5.896\left(1\right)\times {10}^{-7}, {a}_{2}=1.811\left(1\right)\times {10}^{-9}$. Pawley et al. reported that high temperature data of hydrous minerals could be fit by ${\alpha }_{V,T}= {\alpha }_{0}- {\alpha }_{1}{T}^{-\frac{1}{2}}$ with ${\alpha }_{1}=10{\alpha }_{0}$ which further adapted into the Holland and Powell model (Eq. 3)^61,62^.

3 $${V}_{T}= {V}_{0}(1+{a}_{V}^{0}\left(T-{T}_{0}\right)-20{a}_{V}^{0}({T}^\frac{1}{2}-{{T}_{0}}^\frac{1}{2})$$

**Figure 3:**
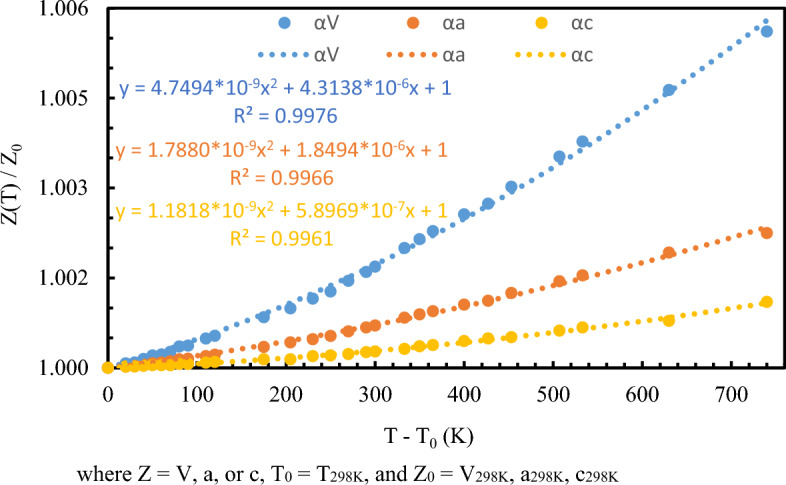
Berman et al. model fit of beryl lattice parameters at various temperatures.

Incorporating the change in beryl unit cell volume gives rise to thermal expansion coefficients, reported as ${a}_{V}^{0}=1.201\left(5\right)\times {10}^{-5}$, corresponds to ${\alpha }_{V,298K}=5.055\left(1\right)\times {10}^{-6}$ and ${\alpha }_{V,1038K}=8.285\left(5\right)\times {10}^{-6}$. The linear axial thermal expansion coefficient can also be derived from Eq. 3, resulting in ${a}_{a}^{0}=4.928\left(1\right)\times {10}^{-6}$, ${a}_{a,298K}^{0}=2.073\left(1\right)\times {10}^{-6}, {a}_{a,1038K}^{0}=3.397\left(2\right)\times {10}^{-6}$; ${a}_{c}^{0}=2.121\left(1\right)\times {10}^{-6}$, ${a}_{a,298K}^{0}=8.922\left(1\right)\times {10}^{-7}, {a}_{a,1038K}^{0}=1.461\left(2\right)\times {10}^{-6}$ for a and c axes, respectively (Figure S7). In the three investigated models, the Berman et al. model gives the best fit for our beryl thermal expansion dataset, while the Holland and Powell model giving a slightly inferior agreement (Fig. 3).

**Figure 4:**
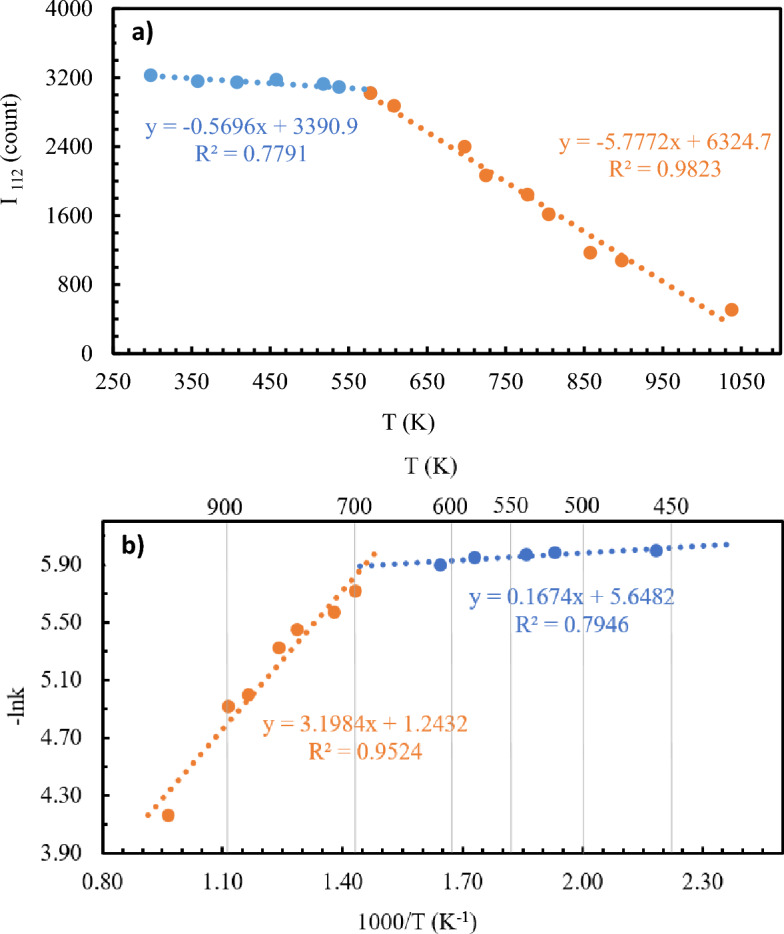
(a) Change in peak intensity of the strong reflection (112) as a function of temperature. (b) Rate constant of the dehydration of beryl plotted in Arrhenius form.

For kinetic interpretations of the collected XRD spectra, the relative analysis workflow is modelled after Sarikaya et al. with appropriate modifications^63^. Using Dioptas, simultaneous background optimization on all XRD patterns was performed^64^. The crystallinity of beryl sample was determined from the absolute intensity of the strongest diffraction peak, (112) reflection^65^. Reflection (112) satisfies the choosing criteria to monitor sample crystallinity as (1) a strong in intensity and easily observable, (2) sufficiently separated from other nearby diffraction peaks, and (3) strong dependency on the water site occupancy factor. Correlation between sample crystallinity and change in temperature is observed with the gradual decrease in (112) reflection intensity, up to 458 K, presumably related to the removal of loosely bound water, followed by a more rapid reduction in crystallinity due to the loss of tightly bound water confined in the nanopore channels and structural degradation at high temperature (Fig. 4a). We further estimate a reaction rate constant, *k*, of the thermal analysis with analogy from the isobaric thermal expansion coefficient using $k= -\frac{1}{I}(\frac{\partial I}{\partial T})$_p_ where $\frac{\partial I}{\partial T}$ is obtained from the slope of the straight line in the plot of intensity at different temperature. The rate constant is plotted in Arrhenius form in Fig. 4b, where the dehydration processes above 458 K can be broken down further into two stages with major loss of water observed at 698 K (Table S2).

### Beryl structure evolution from variable temperature single crystal structure analyses

**Figure 5:**
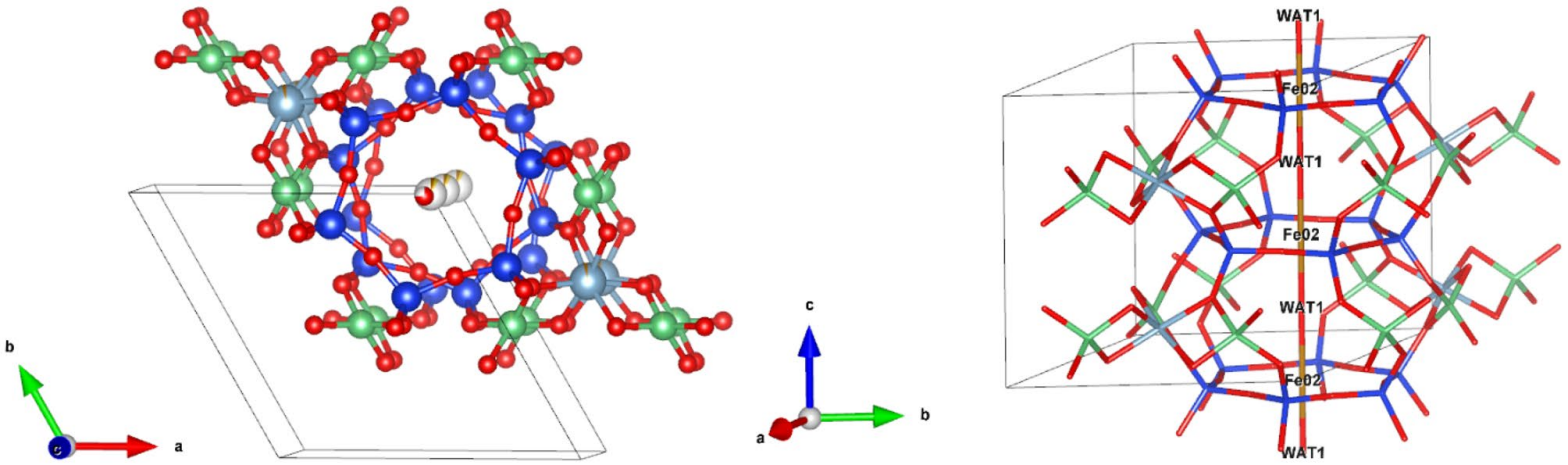
Different orientations of representative beryl crystal structure at 298 K with confined ions Na, Fe (site 2b) and WAT1 (site 2a). Red = oxygen, grey = aluminum, green = beryllium, blue = silicon.

We further propose that the dynamics of confined water in nanopore channel can be probed with a crystallographic model of beryl at each investigated temperature. Standard workflow for data analysis involves obtaining the beryl lattice parameters, using Bruker APEX3, up to the maximum investigated temperature of 1038 K, followed by structural determination in Olex2 with refinement standard values of R_1_% (1.56–4.73), wR_2_% (4.02–12.63), and GooF (0.955–1.293) (Table S3–S6). A representative crystallographic model of beryl at 298 K, with different orientations, is given in Fig. 5, features the Si_6_O_18_ rings, linked together by Be, mixed with trace amounts of Li, and Al, mixed with small amounts of Fe, create a hexagonal packed array of channels that run parallel to the c-axis.

**Figure 6:**
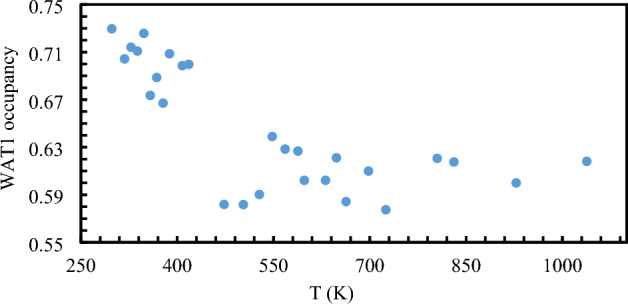
Water occupancy in 2a channel site at various temperatures.

Na and Fe ions were fixed to the microprobe composition while the occupancy of the water WAT1 ion was allowed to vary over the investigated temperature range. The downward trend of WAT1 occupancy at elevated temperatures, illustrates the expected dehydration reaction (Fig. 6). We also observe that the loss of WAT1 occurs in two stages, with the loosely bound water comes off at ~ 450 K while a gradual loss of water occurs at a slower rate, up to 1038 K. The susceptible loss of WAT1 in response to temperature is concomitant with the steady increase in Be–O and Al–O bond length, which expands the void volume, in comparison with insignificant change in Si–O bond length within the silicate ring (Figures S1–S4).

Further literature review on the response of the beryl framework with temperature highlights the work of Hochella and Brown^66^ who determined that the major structural changes with temperature in beryl-type framework structures are expansion of the M site and T2 site and relatively complicated changes in T–O–T, O–T–O, and T–O–M angles in the tetrahedral framework, with Si-, Al–O bonds show no significant changes. The structure can contract along c due to a twisting of the framework partially driven by the M (and T2) polyhedral expansion(s) and the accompanying collapse of O–T–O and T–O–M angles. Large alkali cations like Cs in the C1 site can prevent contraction along c. Ainess and Rossman studied beryl with iron and sodium content comparable to our sample using high temperature IR spectroscopy^41^. They found that above 673 K water that is structurally bound in the beryl channels gradually moves into an unbound state with the characteristics of a gas. The process is fully reversible for both type I and type II water. Dehydration occurs after most of the water is in this unbound state, and channel cations are no longer coordinated to the type II water molecules. These cations can then move to made space for the water transport along the channel. Some anomalies taking place at about 673 K in the T-dependence of the unit cell parameters of beryl and emerald were also observed in the single crystal X-ray diffraction data of Morosin, though full structure refinements were not conducted at high temperatures^19^. Fukuda and Shinoda^67^ studied blue beryl from Otoni, Brazil using polarized IR spectroscopy. Over the temperature range from room to 1073 K where rapid dehydration did not occur, the decrease in band heights for type II water molecules were smaller than those for type I, while band shifts were more predominant for type II water molecules. Significant dehydration was observed at 1123 K. Brown and Mills studied thermal expansion of alkali-rich beryl from the Harding pegmatite using a combination of single crystal X-ray diffraction and IR spectroscopy and determined a different type of behavior^14^. Heat treatment at 1073 K for 72 h had little effect on the occupancies of channel sites with negligible dehydration because the large alkali ions effectively plug the channels.

## Conclusions

In summary, we have demonstrated that it is possible to monitor the behavior of water molecules and identification of water binding environment within the nanopore channel using crystal structure analyses. Our results further confirm that the response of water to temperature, with prominent dehydration kinetics, directly correlates to the expansion of the housing scaffold. This opens up new possibilities in designing scaffolds with targeted binding sites and monitoring the response of the substrate within the nano framework to external stimuli, using advanced crystallography.

### Supplementary Information

Supplementary Information.
