# Supplementary material for: Dehydration kinetics of nanoconfined water in beryl probed by high temperature single crystal synchrotron X-ray diffraction
Source: Sci Rep. 2024 Mar 13;14:6079. doi: 10.1038/s41598-024-53654-4 (PMC10937911; doi:10.1038/s41598-024-53654-4)
Supplement: Supplementary file 1 — Supplementary Information. [file 41598_2024_53654_MOESM1_ESM.docx]

**A crystallographic approach probing dehydration reaction kinetics of nanoconfined water in beryl mineral**

Supplementary information

Phuong Q. H. Nguyen,^1,2*^ Dongzhou Zhang,^1,2^ Jingui Xu,^1,2,3^ Robert T. Downs,^4^ Przemyslaw K. Dera^1*^

1. Hawaii Institute of Geophysics and Planetology, University of Hawaii at Manoa, Honolulu, HI 96822, USA
2. GeoSoilEnviroCARS, University of Chicago, Argonne, IL 60439, USA
3. Key Laboratory for High-Temperature and High-Pressure for the Earth’s Interior, Institute of Geochemistry, Chinese Academy of Sciences. Guiyang, China
4. The University of Arizona, Department of Geosciences, Tucson, AZ 85721-0077, USA

*Correspondence to be addressed to: [nguyenph@hawaii.edu](mailto:nguyenph@hawaii.edu), pdera@hawaii.edu

**Table S1:** The chemical percent compositions of the investigated beryl determined by EPMA

|  | | | | | | | | | |
| --- | --- | --- | --- | --- | --- | --- | --- | --- | --- |
| Na_2_O | Al_2_O_3_ | SiO_2_ | K_2_O | FeO | BeO | Total |  |  |  |
| 0.07(1) | 18.14(22) | 65.47(87) | 0.02(1) | 0.87(6) | 15.43(95) | 100 |  |  |  |
| On the basis of 18 oxygen | | |  |  |  |  |  |  |  |
| Si | Al | Fe | Na | Be | Li** |  |  |  |  |
| 5.89(13) | 1.92(3) | 0.07(1) | 0.01(0) | 3.27(27) |  |  |  |  |  |
| **estimated by charge balance | | | |  |  |  |  |  |  |
| Standard deviations in parentheses are in unit of the last digit | | | | | | |  |  |  |
| Si was assumed with 6.0 atoms per formula unit) | | | | | |  |  |  |  |
| Standards: albite-Cr (Na Kα), kyanite (Si Kα, Al Kα), kspar-OR1 (K Kα), fayalite (Fe Kα) | | | | | | | | | |

**Table S2.** Peak intensity count number of XRD (112) reflection of beryl at various temperature – Dioptas extraction after Pattern Background subtraction (Smooth Width = 0.1, Iterations = 150, Order = 50)^1^

| ***T* (K)** | **1/*T* (10^-3^ K^-1^)** | ***I* (count)** | ***k* (10^-3^ K^-1^)** | ***lnk*** |
| --- | --- | --- | --- | --- |
| 458 | 2.1834 | 3175 | 2.4793 | -5.9998 |
| 518 | 1.9305 | 3125 | 2.5190 | -5.9839 |
| 538 | 1.8587 | 3090 | 2.5475 | -5.9726 |
| 578 | 1.7301 | 3020 | 2.6066 | -5.9497 |
| 608 | 1.6447 | 2872 | 2.7409 | -5.8995 |
| 698 | 1.4327 | 2398 | 3.2827 | -5.7191 |
| 725 | 1.3793 | 2065 | 3.8121 | -5.5696 |
| 778 | 1.2853 | 1842 | 4.2736 | -5.4553 |
| 805 | 1.2422 | 1614 | 4.8773 | -5.3232 |
| 858 | 1.1655 | 1168 | 6.7396 | -4.9997 |
| 898 | 1.1136 | 1077 | 7.3091 | -4.9186 |
| 1038 | 0.9634 | 507 | 15.5264 | -4.1652 |

**Table S3.** Unit-cell parameters and volume of beryl at various temperatures

| *T* (K) | *a* (Å) | *c* (Å) | *α* (^o^) | *γ* (^o^) | *V* (Å^3^) |
| --- | --- | --- | --- | --- | --- |
| 298 | 9.2118(3) | 9.1955(3) | 90 | 120 | 675.76(6) |
| 318 | 9.2120(3) | 9.1957(3) | 90 | 120 | 675.81(6) |
| 328 | 9.2121(3) | 9.1957(3) | 90 | 120 | 675.82(4) |
| 338 | 9.2123(3) | 9.1958(3) | 90 | 120 | 675.86(4) |
| 348 | 9.2125(3) | 9.1959(3) | 90 | 120 | 675.90(4) |
| 358 | 9.2126(3) | 9.1959(3) | 90 | 120 | 675.91(4) |
| 368 | 9.2128(3) | 9.1959(3) | 90 | 120 | 675.94(4) |
| 378 | 9.2131(3) | 9.1961(3) | 90 | 120 | 676.00(4) |
| 388 | 9.2132(3) | 9.1961(3) | 90 | 120 | 676.01(4) |
| 408 | 9.2136(3) | 9.1963(3) | 90 | 120 | 676.09(4) |
| 418 | 9.2138(3) | 9.1964(3) | 90 | 120 | 676.12(4) |
| 473 | 9.2150(3) | 9.1968(3) | 90 | 120 | 676.33(3) |
| 503 | 9.2157(3) | 9.1968(3) | 90 | 120 | 676.43(4) |
| 528 | 9.2162(3) | 9.1973(2) | 90 | 120 | 676.54(3) |
| 548 | 9.2167(3) | 9.1974(3) | 90 | 120 | 676.62(4) |
| 568 | 9.2174(3) | 9.1976(3) | 90 | 120 | 676.74(4) |
| 588 | 9.2180(3) | 9.1979(3) | 90 | 120 | 676.84(4) |
| 598 | 9.2183(3) | 9.1980(3) | 90 | 120 | 676.90(4) |
| 631 | 9.2195(3) | 9.1984(3) | 90 | 120 | 677.11(4) |
| 648 | 9.2200(4) | 9.1988(3) | 90 | 120 | 677.21(5) |
| 663 | 9.2205(4) | 9.1990(4) | 90 | 120 | 677.30(5) |
| 698 | 9.2215(4) | 9.1996(4) | 90 | 120 | 677.49(5) |
| 725 | 9.2221(4) | 9.2000(4) | 90 | 120 | 677.61(5) |
| 751 | 9.2233(3) | 9.2002(3) | 90 | 120 | 677.80(4) |
| 805 | 9.2251(4) | 9.2012(4) | 90 | 120 | 678.14(5) |
| 831 | 9.2260(5) | 9.2017(4) | 90 | 120 | 678.31(6) |
| 928 | 9.2295(6) | 9.2027(5) | 90 | 120 | 678.89(7) |
| 1038 | 9.2325(6) | 9.2056(5) | 90 | 120 | 679.55(7) |

**Table S4.** Selective structure refinement details of beryl at various temperatures

| *T* (K) | *R_int_* (%) | Number total reflection | Number unique reflections | *R_1_* (%) | *wR_2_* (%) | GooF |
| --- | --- | --- | --- | --- | --- | --- |
| 298 | 5.62 | 7912 | 278 | 1.56 | 5.11 | 1.283 |
| 318 | 5.68 | 7929 | 280 | 1.86 | 5.79 | 1.126 |
| 328 | 5.77 | 7913 | 280 | 1.87 | 6.02 | 1.259 |
| 338 | 5.66 | 7930 | 280 | 1.80 | 5.82 | 1.127 |
| 348 | 5.67 | 7905 | 279 | 1.71 | 5.22 | 1.266 |
| 358 | 5.59 | 7926 | 278 | 1.69 | 5.36 | 1.263 |
| 368 | 6.03 | 7911 | 278 | 1.74 | 5.21 | 1.246 |
| 378 | 5.52 | 7914 | 278 | 1.72 | 5.41 | 1.227 |
| 388 | 5.74 | 7912 | 278 | 1.73 | 4.90 | 1.196 |
| 408 | 5.84 | 7928 | 280 | 1.87 | 6.39 | 1.245 |
| 418 | 5.70 | 7929 | 279 | 1.85 | 5.72 | 1.293 |
| 473 | 6.10 | 12415 | 810 | 2.05 | 6.86 | 1.135 |
| 503 | 5.72 | 12434 | 812 | 2.11 | 7.06 | 1.170 |
| 528 | 4.75 | 12361 | 811 | 1.86 | 6.01 | 1.161 |
| 548 | 5.81 | 12413 | 810 | 2.13 | 7.08 | 1.199 |
| 568 | 5.98 | 12436 | 814 | 2.20 | 7.17 | 1.157 |
| 588 | 5.85 | 12440 | 811 | 2.06 | 6.50 | 1.141 |
| 598 | 6.34 | 12438 | 811 | 2.11 | 7.24 | 1.192 |
| 631 | 6.35 | 12446 | 811 | 2.22 | 7.05 | 1.098 |
| 648 | 6.16 | 12491 | 805 | 2.18 | 6.27 | 1.096 |
| 663 | 6.32 | 12464 | 810 | 2.22 | 7.24 | 1.146 |
| 698 | 6.31 | 12520 | 808 | 2.18 | 7.03 | 1.096 |
| 725 | 7.03 | 12560 | 809 | 2.73 | 8.44 | 1.083 |
| 751 | 8.60 | 12503 | 808 | 2.31 | 7.05 | 1.049 |
| 805 | 8.56 | 12436 | 808 | 3.09 | 8.45 | 1.093 |
| 831 | 8.92 | 12004 | 720 | 2.85 | 7.22 | 1.086 |
| 928 | 13.82 | 8604 | 526 | 4.73 | 12.63 | 1.065 |
| 1038 | 13.93 | 11094 | 651 | 4.54 | 11.43 | 0.955 |

**Table S5.** Fractional coordinates and displacement parameters of atoms in beryl at various temperatures

| *T* (K) |  | Si01 | Al01 | Fe01 | O001 | O002 | Be01 | Li01 | WAT1 | Fe02 | Na01 |
| --- | --- | --- | --- | --- | --- | --- | --- | --- | --- | --- | --- |
| 298 | *x* | 0.38762 | 0.66667 | 0.66667 | 0.23597 | 0.49864 | 0.50000 | 0.50000 | 0.00000 | 0.00000 | 0.00000 |
|  | *y* | 0.27167 | 0.33333 | 0.33333 | 0.30881 | 0.35307 | 0.50000 | 0.50000 | 0.00000 | 0.00000 | 0.00000 |
|  | *z* | 0.50000 | 0.75000 | 0.75000 | 0.50000 | 0.64519 | 0.75000 | 0.75000 | 0.25000 | 0.00000 | 0.00000 |
|  | *U_iso_* | 0.001 | 0.002 | 0.002 | 0.007 | 0.004 | 0.004 | 0.004 | 0.092 | 0.014 | 0.014 |
|  |  |  |  |  |  |  |  |  |  |  |  |
| 318 | *x* | 0.38754 | 0.66667 | 0.66667 | 0.23608 | 0.49853 | 0.50000 | 0.50000 | 0.00000 | 0.00000 | 0.00000 |
|  | *y* | 0.27162 | 0.33333 | 0.33333 | 0.30897 | 0.35302 | 0.50000 | 0.50000 | 0.00000 | 0.00000 | 0.00000 |
|  | *z* | 0.50000 | 0.75000 | 0.75000 | 0.50000 | 0.64526 | 0.75000 | 0.75000 | 0.25000 | 0.00000 | 0.00000 |
|  | *U_iso_* | 0.001 | 0.002 | 0.002 | 0.008 | 0.004 | 0.004 | 0.004 | 0.094 | 0.030 | 0.030 |
|  |  |  |  |  |  |  |  |  |  |  |  |
| 328 | *x* | 0.38757 | 0.66667 | 0.66667 | 0.23616 | 0.49864 | 0.50000 | 0.50000 | 0.00000 | 0.00000 | 0.00000 |
|  | *y* | 0.27168 | 0.33333 | 0.33333 | 0.30892 | 0.35307 | 0.50000 | 0.5000 | 0.00000 | 0.00000 | 0.00000 |
|  | *z* | 0.50000 | 0.75000 | 0.75000 | 0.50000 | 0.64518 | 0.75000 | 0.75000 | 0.25000 | 0.00000 | 0.00000 |
|  | *U_iso_* | 0.001 | 0.002 | 0.002 | 0.008 | 0.004 | 0.005 | 0.005 | 0.096 | 0.024 | 0.024 |
|  |  |  |  |  |  |  |  |  |  |  |  |
| 338 | *x* | 0.38753 | 0.66667 | 0.66667 | 0.23617 | 0.49852 | 0.50000 | 0.50000 | 0.00000 | 0.00000 | 0.00000 |
|  | *y* | 0.27164 | 0.33333 | 0.33333 | 0.30903 | 0.35291 | 0.50000 | 0.50000 | 0.00000 | 0.00000 | 0.00000 |
|  | *z* | 0.50000 | 0.27000 | 0.27000 | 0.50000 | 0.64521 | 0.75000 | 0.75000 | 0.25000 | 0.00000 | 0.00000 |
|  | *U_iso_* | 0.001 | 0.002 | 0.002 | 0.008 | 0.004 | 0.004 | 0.004 | 0.099 | 0.029 | 0.029 |
|  |  |  |  |  |  |  |  |  |  |  |  |
| 348 | *x* | 0.38751 | 0.66667 | 0.66667 | 0.23607 | 0.49864 | 0.50000 | 0.50000 | 0.00000 | 0.00000 | 0.00000 |
|  | *y* | 0.27162 | 0.33333 | 0.33333 | 0.30884 | 0.35303 | 0.50000 | 0.50000 | 0.00000 | 0.00000 | 0.00000 |
|  | *z* | 0.50000 | 0.75000 | 0.75000 | 0.50000 | 0.64517 | 0.75000 | 0.75000 | 0.25000 | 0.00000 | 0.00000 |
|  | *U_iso_* | 0.001 | 0.002 | 0.002 | 0.008 | 0.005 | 0.004 | 0.004 | 0.100 | 0.029 | 0.029 |
|  |  |  |  |  |  |  |  |  |  |  |  |
| 358 | *x* | 0.38749 | 0.66667 | 0.66667 | 0.23620 | 0.49857 | 0.50000 | 0.50000 | 0.00000 | 0.00000 | 0.00000 |
|  | *y* | 0.27165 | 0.33333 | 0.33333 | 0.30901 | 0.35299 | 0.50000 | 0.50000 | 0.00000 | 0.00000 | 0.00000 |
|  | *z* | 0.50000 | 0.75000 | 0.75000 | 0.50000 | 0.64521 | 0.75000 | 0.75000 | 0.25000 | 0.00000 | 0.00000 |
|  | *U_iso_* | 0.001 | 0.002 | 0.002 | 0.008 | 0.004 | 0.004 | 0.004 | 0.095 | 0.027 | 0.027 |
|  |  |  |  |  |  |  |  |  |  |  |  |
| 368 | *x* | 0.38748 | 0.66667 | 0.66667 | 0.23612 | 0.49858 | 0.50000 | 0.50000 | 0.00000 | 0.00000 | 0.00000 |
|  | *y* | 0.27160 | 0.33333 | 0.33333 | 0.30889 | 0.35299 | 0.50000 | 0.50000 | 0.00000 | 0.00000 | 0.00000 |
|  | *z* | 0.50000 | 0.75000 | 0.75000 | 0.50000 | 0.64524 | 0.75000 | 0.75000 | 0.25000 | 0.00000 | 0.00000 |
|  | *U_iso_* | 0.001 | 0.002 | 0.002 | 0.008 | 0.005 | 0.004 | 0.004 | 0.097 | 0.024 | 0.024 |
|  |  |  |  |  |  |  |  |  |  |  |  |
| 378 | *x* | 0.38743 | 0.66667 | 0.66667 | 0.23610 | 0.49855 | 0.50000 | 0.50000 | 0.00000 | 0.00000 | 0.00000 |
|  | *y* | 0.27161 | 0.33333 | 0.33333 | 0.30895 | 0.35299 | 0.50000 | 0.50000 | 0.00000 | 0.00000 | 0.00000 |
|  | *z* | 0.50000 | 0.75000 | 0.75000 | 0.50000 | 0.64522 | 0.75000 | 0.75000 | 0.25000 | 0.00000 | 0.00000 |
|  | *U_iso_* | 0.002 | 0.002 | 0.002 | 0.009 | 0.005 | 0.005 | 0.005 | 0.098 | 0.024 | 0.024 |
|  |  |  |  |  |  |  |  |  |  |  |  |
| 388 | *x* | 0.38742 | 0.66667 | 0.66667 | 0.23613 | 0.49855 | 0.50000 | 0.50000 | 0.00000 | 0.00000 | 0.00000 |
|  | *y* | 0.27158 | 0.33333 | 0.33333 | 0.30890 | 0.35301 | 0.50000 | 0.50000 | 0.00000 | 0.00000 | 0.00000 |
|  | *z* | 0.50000 | 0.75000 | 0.75000 | 0.50000 | 0.64524 | 0.75000 | 0.75000 | 0.25000 | 0.00000 | 0.00000 |
|  | *U_iso_* | 0.002 | 0.003 | 0.003 | 0.009 | 0.005 | 0.005 | 0.005 | 0.101 | 0.026 | 0.026 |
|  |  |  |  |  |  |  |  |  |  |  |  |
| 408 | *x* | 0.38745 | 0.66667 | 0.66667 | 0.23609 | 0.49852 | 0.50000 | 0.50000 | 0.00000 | 0.00000 | 0.00000 |
|  | *y* | 0.27164 | 0.33333 | 0.33333 | 0.30882 | 0.35291 | 0.50000 | 0.50000 | 0.00000 | 0.00000 | 0.00000 |
|  | *z* | 0.50000 | 0.75000 | 0.75000 | 0.50000 | 0.64519 | 0.75000 | 0.75000 | 0.25000 | 0.00000 | 0.00000 |
|  | *U_iso_* | 0.002 | 0.003 | 0.003 | 0.009 | 0.005 | 0.005 | 0.005 | 0.108 | 0.038 | 0.038 |
|  |  |  |  |  |  |  |  |  |  |  |  |
| 418 | *x* | 0.38739 | 0.66667 | 0.66667 | 0.23607 | 0.49853 | 0.50000 | 0.50000 | 0.00000 | 0.00000 | 0.00000 |
|  | *y* | 0.27156 | 0.33333 | 0.33333 | 0.30889 | 0.35294 | 0.50000 | 0.50000 | 0.00000 | 0.00000 | 0.00000 |
|  | *z* | 0.50000 | 0.75000 | 0.75000 | 0.50000 | 0.64518 | 0.75000 | 0.75000 | 0.25000 | 0.00000 | 0.00000 |
|  | *U_iso_* | 0.002 | 0.003 | 0.003 | 0.010 | 0.005 | 0.005 | 0.005 | 0.1110 | 0.029 | 0.029 |
|  |  |  |  |  |  |  |  |  |  |  |  |
| 473 | *x* | 0.38729 | 0.66667 | 0.66667 | 0.23586 | 0.49842 | 0.50000 | 0.50000 | 0.00000 | 0.00000 | 0.00000 |
|  | *y* | 0.27145 | 0.33333 | 0.33333 | 0.30910 | 0.35297 | 0.50000 | 0.50000 | 0.00000 | 0.00000 | 0.00000 |
|  | *z* | 0.50000 | 0.75000 | 0.75000 | 0.50000 | 0.64518 | 0.75000 | 0.75000 | 0.25000 | 0.00000 | 0.00000 |
|  | *U_iso_* | 0.003 | 0.004 | 0.004 | 0.011 | 0.007 | 0.006 | 0.006 | 0.103 | 0.036 | 0.036 |
|  |  |  |  |  |  |  |  |  |  |  |  |
| 503 | *x* | 0.38727 | 0.66667 | 0.66667 | 0.23585 | 0.49843 | 0.50000 | 0.50000 | 0.00000 | 0.00000 | 0.00000 |
|  | *y* | 0.27143 | 0.33333 | 0.33333 | 0.30906 | 0.35296 | 0.50000 | 0.50000 | 0.00000 | 0.00000 | 0.00000 |
|  | *z* | 0.50000 | 0.75000 | 0.75000 | 0.50000 | 0.64510 | 0.75000 | 0.75000 | 0.25000 | 0.00000 | 0.00000 |
|  | *U_iso_* | 0.003 | 0.004 | 0.004 | 0.011 | 0.007 | 0.006 | 0.006 | 0.110 | 0.036 | 0.036 |
|  |  |  |  |  |  |  |  |  |  |  |  |
| 528 | *x* | 0.38723 | 0.66667 | 0.66667 | 0.23585 | 0.49839 | 0.50000 | 0.50000 | 0.00000 | 0.00000 | 0.00000 |
|  | *y* | 0.27141 | 0.33333 | 0.33333 | 0.30894 | 0.35293 | 0.50000 | 0.50000 | 0.00000 | 0.00000 | 0.00000 |
|  | *z* | 0.50000 | 0.75000 | 0.75000 | 0.50000 | 0.64509 | 0.75000 | 0.75000 | 0.25000 | 0.00000 | 0.00000 |
|  | *U_iso_* | 0.003 | 0.004 | 0.004 | 0.012 | 0.007 | 0.007 | 0.007 | 0.107 | 0.034 | 0.034 |
|  |  |  |  |  |  |  |  |  |  |  |  |
| 548 | *x* | 0.38720 | 0.66667 | 0.66667 | 0.23595 | 0.49833 | 0.50000 | 0.50000 | 0.00000 | 0.00000 | 0.00000 |
|  | *y* | 0.27137 | 0.33333 | 0.33333 | 0.30908 | 0.35291 | 0.50000 | 0.50000 | 0.00000 | 0.00000 | 0.00000 |
|  | *z* | 0.50000 | 0.75000 | 0.75000 | 0.50000 | 0.64520 | 0.75000 | 0.75000 | 0.25000 | 0.00000 | 0.00000 |
|  | *U_iso_* | 0.004 | 0.005 | 0.005 | 0.013 | 0.008 | 0.007 | 0.007 | 0.130 | 0.051 | 0.051 |
|  |  |  |  |  |  |  |  |  |  |  |  |
| 568 | *x* | 0.38715 | 0.66667 | 0.66667 | 0.23586 | 0.49826 | 0.50000 | 0.50000 | 0.00000 | 0.00000 | 0.00000 |
|  | *y* | 0.27134 | 0.33333 | 0.33333 | 0.30902 | 0.35286 | 0.50000 | 0.50000 | 0.00000 | 0.00000 | 0.00000 |
|  | *z* | 0.50000 | 0.75000 | 0.75000 | 0.50000 | 0.64526 | 0.75000 | 0.75000 | 0.25000 | 0.00000 | 0.00000 |
|  | *U_iso_* | 0.004 | 0.005 | 0.005 | 0.013 | 0.008 | 0.007 | 0.007 | 0.123 | 0.056 | 0.056 |
|  |  |  |  |  |  |  |  |  |  |  |  |
| 588 | *x* | 0.38715 | 0.66667 | 0.66667 | 0.23583 | 0.49829 | 0.50000 | 0.50000 | 0.00000 | 0.00000 | 0.00000 |
|  | *y* | 0.27133 | 0.33333 | 0.33333 | 0.30897 | 0.35291 | 0.50000 | 0.50000 | 0.00000 | 0.00000 | 0.00000 |
|  | *z* | 0.50000 | 0.75000 | 0.75000 | 0.50000 | 0.64515 | 0.75000 | 0.75000 | 0.25000 | 0.00000 | 0.00000 |
|  | *U_iso_* | 0.004 | 0.005 | 0.005 | 0.014 | 0.008 | 0.008 | 0.008 | 0.135 | 0.065 | 0.065 |
|  |  |  |  |  |  |  |  |  |  |  |  |
| 598 | *x* | 0.38709 | 0.66667 | 0.66667 | 0.23578 | 0.49826 | 0.50000 | 0.50000 | 0.00000 | 0.00000 | 0.00000 |
|  | *y* | 0.27130 | 0.33333 | 0.33333 | 0.30896 | 0.35285 | 0.50000 | 0.50000 | 0.00000 | 0.00000 | 0.00000 |
|  | *z* | 0.50000 | 0.75000 | 0.75000 | 0.50000 | 0.64511 | 0.75000 | 0.75000 | 0.25000 | 0.00000 | 0.00000 |
|  | *U_iso_* | 0.004 | 0.005 | 0.005 | 0.014 | 0.009 | 0.008 | 0.008 | 0.121 | 0.053 | 0.053 |
|  |  |  |  |  |  |  |  |  |  |  |  |
| 631 | *x* | 0.38704 | 0.66667 | 0.66667 | 0.23579 | 0.49823 | 0.50000 | 0.50000 | 0.00000 | 0.00000 | 0.00000 |
|  | *y* | 0.27127 | 0.33333 | 0.33333 | 0.30895 | 0.35280 | 0.50000 | 0.50000 | 0.00000 | 0.00000 | 0.00000 |
|  | *z* | 0.50000 | 0.75000 | 0.75000 | 0.50000 | 0.64513 | 0.75000 | 0.75000 | 0.25000 | 0.00000 | 0.00000 |
|  | *U_iso_* | 0.005 | 0.006 | 0.006 | 0.015 | 0.010 | 0.009 | 0.009 | 0.133 | 0.054 | 0.054 |
|  |  |  |  |  |  |  |  |  |  |  |  |
| 648 | *x* | 0.38701 | 0.66667 | 0.66667 | 0.23586 | 0.49822 | 0.50000 | 0.50000 | 0.00000 | 0.00000 | 0.00000 |
|  | *y* | 0.27124 | 0.33333 | 0.33333 | 0.30892 | 0.35281 | 0.50000 | 0.50000 | 0.00000 | 0.00000 | 0.00000 |
|  | *z* | 0.50000 | 0.75000 | 0.75000 | 0.50000 | 0.64514 | 0.75000 | 0.75000 | 0.25000 | 0.00000 | 0.00000 |
|  | *U_iso_* | 0.005 | 0.006 | 0.006 | 0.015 | 0.010 | 0.009 | 0.009 | 0.144 | 0.059 | 0.059 |
|  |  |  |  |  |  |  |  |  |  |  |  |
| 663 | *x* | 0.38697 | 0.66667 | 0.66667 | 0.23583 | 0.49816 | 0.50000 | 0.50000 | 0.00000 | 0.00000 | 0.00000 |
|  | *y* | 0.27122 | 0.33333 | 0.33333 | 0.30893 | 0.35272 | 0.50000 | 0.50000 | 0.00000 | 0.00000 | 0.00000 |
|  | *z* | 0.50000 | 0.75000 | 0.75000 | 0.50000 | 0.64510 | 0.75000 | 0.75000 | 0.25000 | 0.00000 | 0.00000 |
|  | *U_iso_* | 0.005 | 0.006 | 0.006 | 0.016 | 0.010 | 0.009 | 0.009 | 0.133 | 0.054 | 0.054 |
|  |  |  |  |  |  |  |  |  |  |  |  |
| 698 | *x* | 0.38696 | 0.66667 | 0.66667 | 0.23582 | 0.49811 | 0.50000 | 0.50000 | 0.00000 | 0.00000 | 0.00000 |
|  | *y* | 0.27122 | 0.33333 | 0.33333 | 0.30894 | 0.35268 | 0.50000 | 0.50000 | 0.00000 | 0.00000 | 0.00000 |
|  | *z* | 0.50000 | 0.75000 | 0.75000 | 0.50000 | 0.64512 | 0.75000 | 0.75000 | 0.25000 | 0.00000 | 0.00000 |
|  | *U_iso_* | 0.005 | 0.007 | 0.007 | 0.016 | 0.011 | 0.010 | 0.010 | 0.144 | 0.055 | 0.055 |
|  |  |  |  |  |  |  |  |  |  |  |  |
| 725 | *x* | 0.38693 | 0.66667 | 0.66667 | 0.23581 | 0.49814 | 0.50000 | 0.50000 | 0.00000 | 0.00000 | 0.00000 |
|  | *y* | 0.27123 | 0.33333 | 0.33333 | 0.30886 | 0.35267 | 0.50000 | 0.50000 | 0.00000 | 0.00000 | 0.00000 |
|  | *z* | 0.50000 | 0.75000 | 0.75000 | 0.50000 | 0.64509 | 0.75000 | 0.75000 | 0.25000 | 0.00000 | 0.00000 |
|  | *U_iso_* | 0.006 | 0.007 | 0.007 | 0.017 | 0.011 | 0.011 | 0.011 | 0.133 | 0.068 | 0.068 |
|  |  |  |  |  |  |  |  |  |  |  |  |
| 751 | *x* | 0.38682 | 0.66667 | 0.66667 | 0.23579 | 0.49812 | 0.50000 | 0.50000 | 0.00000 | 0.00000 | 0.00000 |
|  | *y* | 0.27114 | 0.33333 | 0.33333 | 0.30882 | 0.35271 | 0.50000 | 0.50000 | 0.00000 | 0.00000 | 0.00000 |
|  | *z* | 0.50000 | 0.75000 | 0.75000 | 0.50000 | 0.64497 | 0.75000 | 0.75000 | 0.25000 | 0.00000 | 0.00000 |
|  | *U_iso_* | 0.006 | 0.007 | 0.007 | 0.017 | 0.011 | 0.010 | 0.010 | 0.139 | 0.060 | 0.060 |
| 805 | *x* | 0.38676 | 0.66667 | 0.66667 | 0.23588 | 0.49802 | 0.50000 | 0.50000 | 0.00000 | 0.00000 | 0.00000 |
|  | *y* | 0.27111 | 0.33333 | 0.33333 | 0.30899 | 0.35262 | 0.50000 | 0.50000 | 0.00000 | 0.00000 | 0.00000 |
|  | *z* | 0.50000 | 0.75000 | 0.75000 | 0.50000 | 0.64500 | 0.75000 | 0.75000 | 0.25000 | 0.00000 | 0.00000 |
|  | *U_iso_* | 0.007 | 0.008 | 0.008 | 0.019 | 0.013 | 0.012 | 0.012 | 0.157 | 0.057 | 0.057 |
| 831 | *x* | 0.38670 | 0.66667 | 0.66667 | 0.23577 | 0.49795 | 0.50000 | 0.50000 | 0.00000 | 0.00000 | 0.00000 |
|  | *y* | 0.27104 | 0.33333 | 0.33333 | 0.30876 | 0.35256 | 0.50000 | 0.50000 | 0.00000 | 0.00000 | 0.00000 |
|  | *z* | 0.50000 | 0.75000 | 0.75000 | 0.50000 | 0.64501 | 0.75000 | 0.75000 | 0.25000 | 0.00000 | 0.00000 |
|  | *U_iso_* | 0.007 | 0.009 | 0.009 | 0.050 | 0.013 | 0.012 | 0.012 | 0.151 | 0.060 | 0.060 |
| 928 | *x* | 0.38655 | 0.66667 | 0.66667 | 0.23556 | 0.49784 | 0.50000 | 0.50000 | 0.00000 | 0.00000 | 0.00000 |
|  | *y* | 0.27105 | 0.33333 | 0.33333 | 0.30846 | 0.35239 | 0.50000 | 0.50000 | 0.00000 | 0.00000 | 0.00000 |
|  | *z* | 0.50000 | 0.75000 | 0.75000 | 0.50000 | 0.64510 | 0.75000 | 0.75000 | 0.25000 | 0.00000 | 0.00000 |
|  | *U_iso_* | 0.008 | 0.010 | 0.010 | 0.022 | 0.015 | 0.014 | 0.014 | 0.147 | 0.063 | 0.063 |
| 1038 | *x* | 0.38644 | 0.66667 | 0.66667 | 0.23544 | 0.49768 | 0.50000 | 0.50000 | 0.00000 | 0.00000 | 0.00000 |
|  | *y* | 0.27090 | 0.33333 | 0.33333 | 0.30821 | 0.35229 | 0.50000 | 0.50000 | 0.00000 | 0.00000 | 0.00000 |
|  | *z* | 0.50000 | 0.75000 | 0.75000 | 0.50000 | 0.64491 | 0.75000 | 0.75000 | 0.25000 | 0.00000 | 0.00000 |
|  | *U_iso_* | 0.010 | 0.012 | 0.012 | 0.025 | 0.017 | 0.016 | 0.016 | 0.190 | 0.072 | 0.072 |

**Table S6.** Selected interatomic distances of beryl at various temperatures

| *T* (K) | Be01-O002 (Å) | Si01-O001 (Å) | Si01-O002 (Å) | Al02-O002 (Å) |
| --- | --- | --- | --- | --- |
| 298 | 1.6566(10) | 1.5958(17) | 1.6197(9) | 1.9077(11) |
| 318 | 1.6564(12) | 1.595(2) | 1.6200(10) | 1.9080(12) |
| 328 | 1.6566(12) | 1.594(2) | 1.6199(10) | 1.9077(12) |
| 338 | 1.6576(12) | 1.595(3) | 1.6194(10) | 1.9078(12) |
| 348 | 1.6570(12) | 1.594(2) | 1.6202(10) | 1.9077(12) |
| 358 | 1.6569(10) | 1.5940(17) | 1.6202(9) | 1.9079(11) |
| 368 | 1.6568(11) | 1.5942(18) | 1.6206(9) | 1.9077(11) |
| 378 | 1.6568(11) | 1.5944(17) | 1.6205(9) | 1.9081(11) |
| 388 | 1.6562(11) | 1.5942(19) | 1.6210(9) | 1.9082(11) |
| 408 | 1.6575(12) | 1.594(2) | 1.6200(10) | 1.9082(12) |
| 418 | 1.6574(11) | 1.5943(19) | 1.6204(9) | 1.9083(12) |
| 473 | 1.6570(5) | 1.5974(8) | 1.6206(5) | 1.9095(5) |
| 503 | 1.6576(5) | 1.5973(9) | 1.6202(5) | 1.9099(5) |
| 528 | 1.6578(4) | 1.5964(8) | 1.6202(4) | 1.9102(5) |
| 548 | 1.6572(5) | 1.5964(9) | 1.6210(5) | 1.9102(5) |
| 568 | 1.6571(5) | 1.5967(9) | 1.6214(5) | 1.9103(5) |
| 588 | 1.6575(5) | 1.5969(8) | 1.6209(5) | 1.9109(5) |
| 598 | 1.6581(5) | 1.5970(9) | 1.6206(5) | 1.9111(5) |
| 631 | 1.6584(6) | 1.5967(9) | 1.6210(6) | 1.9113(6) |
| 648 | 1.6584(5) | 1.5960(9) | 1.6213(5) | 1.9114(5) |
| 663 | 1.6591(6) | 1.5961(9) | 1.6208(6) | 1.9118(6) |
| 698 | 1.6593(6) | 1.5963(10) | 1.6209(6) | 1.9121(6) |
| 725 | 1.6597(7) | 1.5957(11) | 1.6209(6) | 1.9121(7) |
| 751 | 1.6601(6) | 1.5955(10) | 1.6207(6) | 1.9130(6) |
| 805 | 1.6606(8) | 1.5956(13) | 1.6209(7) | 1.9136(8) |
| 831 | 1.6610(8) | 1.5952(13) | 1.6208(7) | 1.9142(8) |
| 928 | 1.6617(17) | 1.594(3) | 1.6219(15) | 1.9144(17) |
| 1038 | 1.6634(14) | 1.594(3) | 1.6209(13) | 1.9167(14) |

**Table S7.** Raman shift (cm^-1^) of the investigated beryl with band assignment based on reported literature values [2-15]

| **Absorption (cm^-1^)** | **Band assignments** |
| --- | --- |
| 254 | E_1g_ |
| 325 | ν_satelitte_, ring/E_2g_ + A_g_ overlap |
| 400 | ν_satelitte_, ring/A_g_ + E_2g_ overlap |
| 421 | E_2g_ |
| 448 | E_2g_ (O-Si-O) |
| 529 | ν(Al-O), symmetric ring deformation/E_1g_ |
| 625 | A_g_, characteristic for ring structures |
| 683 | ν(Be-O), symmetric ring deformation/E_1g_ |
| 772 | ν(Be-O), E_1g_ + E_2g_ overlap |
| 924 | ν(Si-O), E_2g_ + E_1g_ overlap |
| 1014 | ν(Si-O), ring/E_1g_ |
| 1069 | ν(Si-O), ring/A_g_ |
| 1245 | ν(Si-O), ring/E_2g_ |
| 3609 | ν_1_ H_2_O type I |

**Figure S1.** Be01-O002 bond distance as a function of temperature

**Figure S2.** Al02-O004 bond distance as a function of temperature

**Figure S3.** Si01-O002 bond distance as a function of temperature

**Figure S4.** Si01-O001 bond distance as a function of temperature


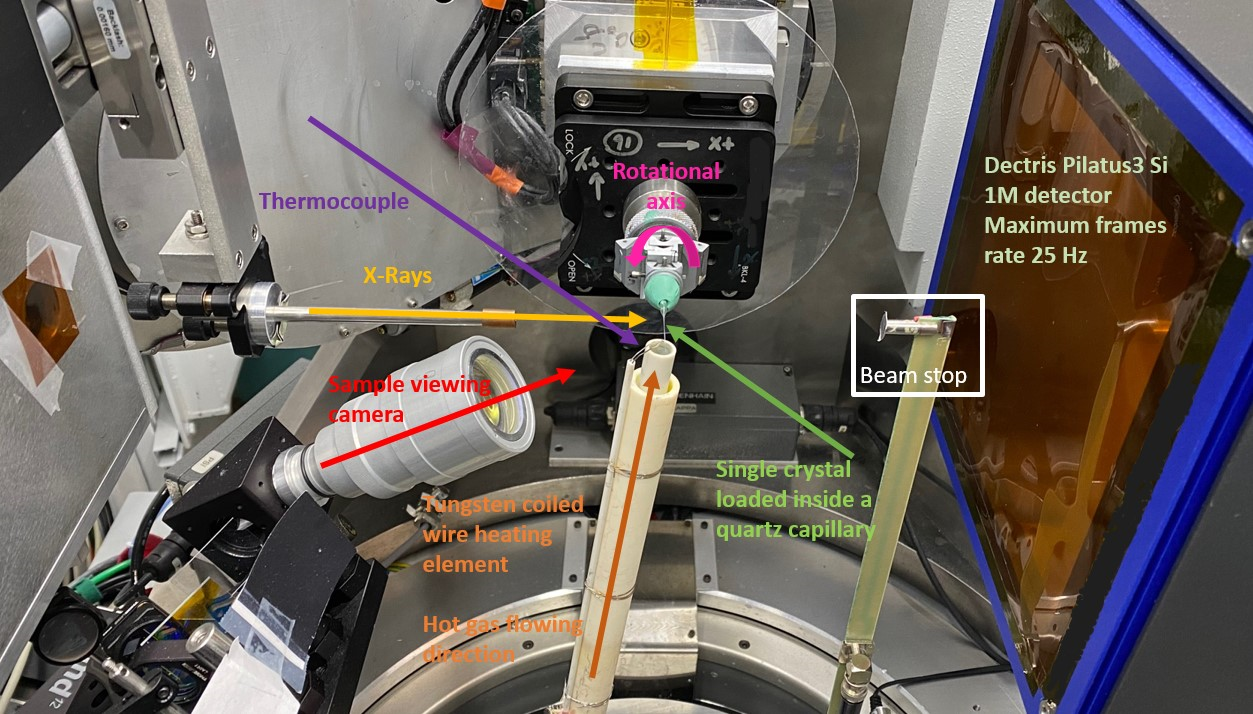


**Figure S5.** Apparatus for in situ synchrotron data collection of single crystal beryl at elevated temperatures, Advanced Photon Source, GSECARS 13 BM-C

where Z = V, a, or c, and Z_0_ = V_298K_, a_298K_, or c_298K_

**Figure S6.** Least-square fit of beryl lattice parameters at various temperatures

**Figure S7.** Holland and Powell model fit of beryl lattice parameters at various temperatures

**Figure S8.** Raman spectra of investigated beryl. Laser is parallel to a* (100). Fiducial mark perpendicular to laser is parallel to c (001). Direction of polarization of laser relative to fiducial mark is 0^o^

**Figure S9.** Raman spectra of investigated beryl. Laser is parallel to a* (100). Fiducial mark perpendicular to laser is parallel to c (001). Direction of polarization of laser is 90^o^ counterclockwise relative to fiducial mark


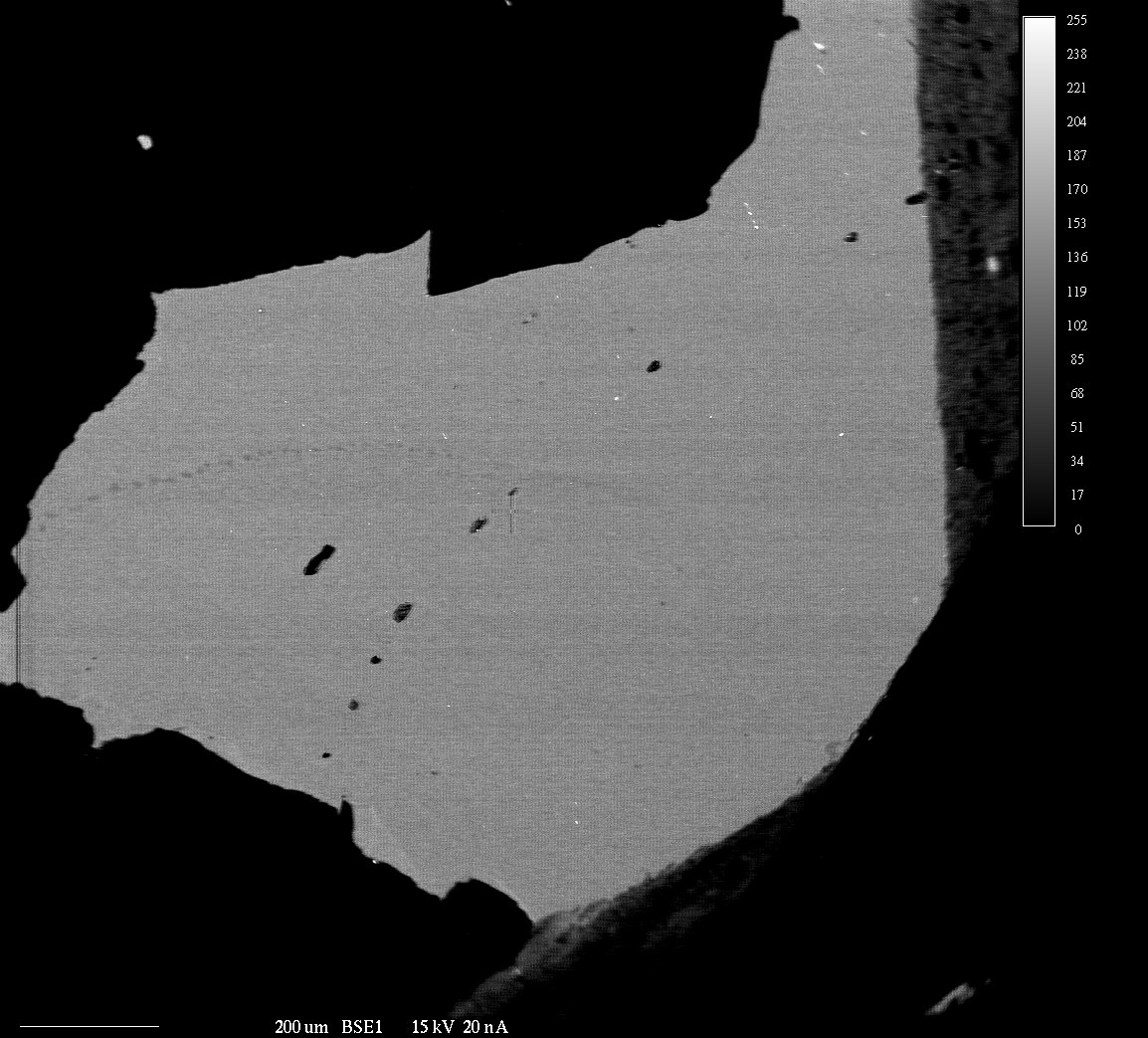


**Figure S10.** Back-scattered electrons (BSE) image of a beryl grain used for EPMA measurements

**Figure S11.** Temperature feedback from thermocouple placed at heating tube exit against temperature determined from thermal expansion of gold flake


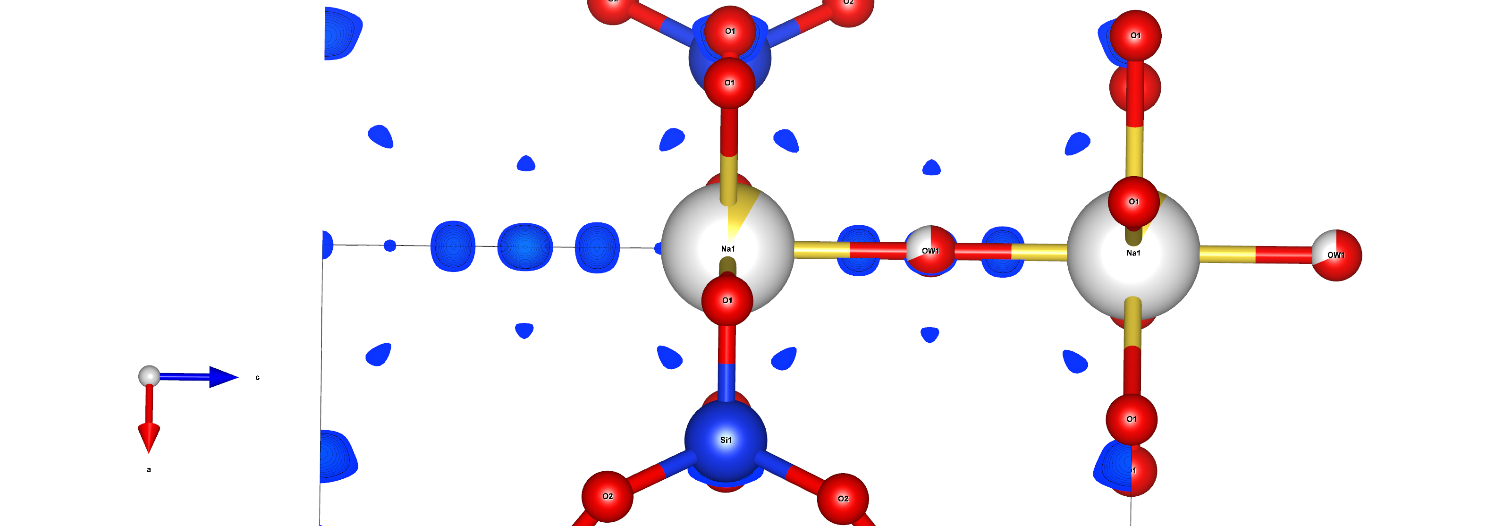


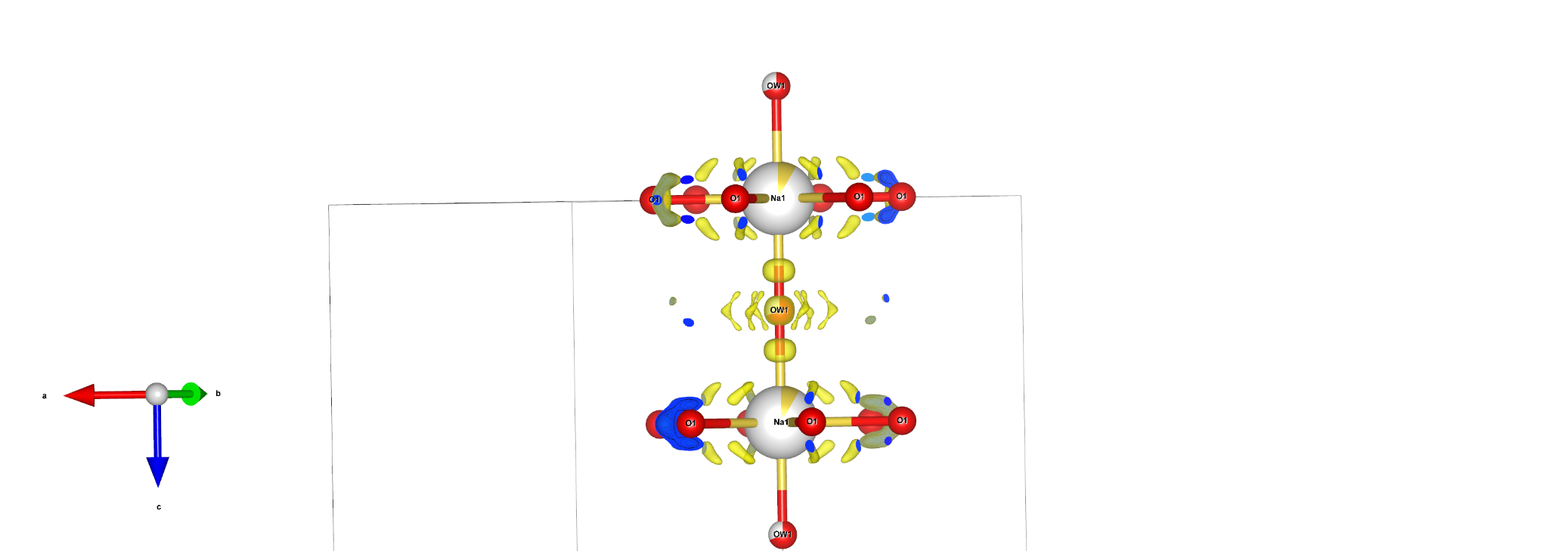


**Figure S12.** Difference Fourier maps indicating distribution of difference electron density (F_obs_-F_calc_) around the oxygen atom of the water molecule in the beryl channel at T=294K. Top and bottom figures indicate different viewing orientations.

Reference

1. Prescher, C., Prakapenka, V.B., DIOPTAS: a program for reduction of two-dimensional X-ray diffraction data and data exploration. ***High Press. Res.***, 35(3) (2015), 223-230
2. B. Charoy, P. de Donato, O. Barres, C. Pinto-Choleo, Am. Mineral. 81 (1996) 395
3. J. T. Kloprogge. R. L. Frost, Spectrochim. Acta A 56 (2000) 501
4. H. Hagemann, A. Lucken, H. Bill, J. Gysler-Sanz, H. A. Stalder, Phys. Chem. Miner. 17 (1990) 395
5. A. M. Hofmeister, T. C. Hoering, D. Virco, Phys. Chem. Miner. 14 (1987) 205
6. W. P. Griffith, Advances in the Raman and infrared spectroscopy of minerals in: R. J. H. Clark, R. E. Hester (Eds.). Spectroscopy of Inorganic-based Materials, Wiley, London, 1987, p. 119
7. D. M. Adams, I. R. Gardner, J. Chem. Soc., Dalton Trans. 14 (1974) 1502
8. E. O’Bannon III, Q. Williams, Phys. Chem. Miner. 43 (2016) 671
9. M. Lodzinski, M. Sitarz, K. Stec, M. Kozanecki, Z. Fojud, S. Jurga, J. Mol. Struct. 744-747 (2005) 1005
10. M. N. Taran, M. D. Dyar, V. M. Khomenko, Phys. Chem. Miner. 45 (2018) 489
11. B. Kolesov, Phys. Chem. Miner. 35 (2008) 271
12. Q. Suo, P. Shen, Y. Luo, C. Li, H. Feng, C. Cao, H. Pan, Y. Bai, Minerals 12 (2022) 450
13. J. Fridrichova, P. Bacik, V. Bizovska, E. Libowitzky, R. Skoda, P. Uher, D. Ozdin, M. Stevko, Phys. Chem. Miner. 43 (2016) 419
14. G. C. Hwang, H. Kim, Y. Lee, J. Miner. Soc. Korea, 30 (2017) 83
15. B. A Kolesov, C. A. Geiger, Phys. Chem. Miner. 27 (2000) 557
